# Supplementary material for: Validated Tools for Assessing Anxiety and Depression in Nurses: A Systematic Review
Source: Int J Environ Res Public Health. 2025 Nov 13;22(11):1714. doi: 10.3390/ijerph22111714 (PMC12652131; doi:10.3390/ijerph22111714)
Supplement: Supplementary file 1 [file ijerph-22-01714-s001.zip › ijerph-3876788-supplementary.pdf]

Supplementary material

Table S1. Quality/Risk of Bias Appraisal (JBI). Included Studies (n=22)

Study (first author and year)	Design	JBI checklist used	Sampling	Confounders addressed	Outcome measurement	Statistical adjustment	Response rate	Overall risk of bias	Key notes / limitations
**Chowdhury, S. R., et al. (2022) [14]**	Cross-sectional	Analytical cross-sectional	Non-probability / convenience (not specified)	Partial	Self-report (e.g., BMS + WPVS + NAQ + SIJS-5)	Limited	NR	Moderate	cross-sectional design limits causal inference; self-report measures; potential information and common-method bias; non-probability sampling; limited generalizability; context-specific to Bangladesh
**Potter G., et al (2021) [15]**	Observational (cohort/longitudinal)	Cohort	Non-probability / convenience (not specified)	Partial (longitudinal models)	Self-report (e.g., OLBI)	Yes (longitudinal models)	NR	Moderate	risk of attrition and residual confounding despite adjustment; self-report measures; potential information and common-method bias; non-probability sampling; limited

									generalizability; context-specific to Germany
**Bock C., et al. (2020) [16]**	Cross-sectional	Analytical cross-sectional	Non-probability / convenience (not specified)	Partial	Self-report (e.g., WFZA)	Limited	NR	Moderate	cross-sectional design limits causal inference; self-report measures; potential information and common-method bias; non-probability sampling; limited generalizability; context-specific to Germany
**Mensinger J.L., et al (2022) [17]**	Cross-sectional	Analytical cross-sectional	Non-probability / convenience (not specified)	Partial	Self-report (e.g., ISI + IES-R)	Limited	NR	Moderate	cross-sectional design limits causal inference; self-report measures; potential information and common-method bias; non-probability sampling; limited generalizability; context-specific to USA

**Ding C., et al. (2023) [18]**	Cross-sectional	Analytical cross-sectional	Non-probability / convenience (not specified)	Partial	Self-report (e.g., WVS + SQS + PDQ-5 + MSPSS + CD-RISC-10 + Three item loneliness scale)	Limited	NR	Moderate	cross-sectional design limits causal inference; self-report measures; potential information and common-method bias; non-probability sampling; limited generalizability; context-specific to China; PSM/multivariable adjustment used; residual confounding remains
**Marsden K.M., et al. (2022) [19]**	Observational (cohort/longitudinal)	Cohort	Non-probability / convenience (not specified)	Partial (longitudinal models)	Self-report (e.g., ISI + IES-R)	Yes (longitudinal models)	NR	Moderate	risk of attrition and residual confounding despite adjustment; self-report measures; potential information and common-method bias; non-probability sampling; limited generalizability; context-specific to Australia

**Cook A., et al. (2021) [20]**	Cross-sectional	Analytical cross-sectional	Convenience sampling	Partial	Self-report (e.g., MBI)	Limited	NR	Moderate	cross-sectional design limits causal inference; self-report measures; potential information and common-method bias; non-probability sampling; limited generalizability; context-specific to USA
**Zanjani M. E., et al. (2021) [21]**	Cross-sectional	Analytical cross-sectional	Non-probability / convenience (not specified)	Partial	Self-report (e.g., SCAS-R + PSS + NIRTQ-2)	Limited	NR	Moderate	cross-sectional design limits causal inference; self-report measures; potential information and common-method bias; non-probability sampling; limited generalizability; context-specific to Australia
**Marin A.M., et al. (2020) [22]**	Descriptive cross-sectional	Analytical cross-sectional	Non-probability / convenience (not specified)	Partial	Self-report (e.g., DECORE + NASA-TLX)	Limited	NR	Moderate	cross-sectional design limits causal inference; self-report measures; potential information and common-method bias; non-

									probability sampling; limited generalizability; context-specific to Spain
**Kowalczuk K, et al. (2020) [23]**	Cross-sectional	Analytical cross-sectional	Non-probability / convenience (not specified)	Partial	Self-report (e.g., SWEQ)	Limited	NR	Moderate	cross-sectional design limits causal inference; self-report measures; potential information and common-method bias; non-probability sampling; limited generalizability; context-specific to Poland
**Łopatkiewicz A., et al. (2023) [24]**	Cross-sectional	Analytical cross-sectional	Non-probability / convenience (not specified)	Partial	Self-report (e.g., MBI)	Limited	NR	Moderate	cross-sectional design limits causal inference; self-report measures; potential information and common-method bias; non-probability sampling; limited generalizability; context-specific to Poland, Germany, Italy, Czech Republic, Slovakia, Hungary

**Seabra P. R. C., et al. (2019) [25]**	Cross-sectional	Analytical cross-sectional	Non-probability / convenience (not specified)	Partial	Self-report (e.g., -)	Limited	NR	Moderate	cross-sectional design limits causal inference; self-report measures; potential information and common-method bias; non-probability sampling; limited generalizability; context-specific to Portugal
**Ariapooran S., et al. (2018) [26]**	Comparative cross-sectional	Analytical cross-sectional	Non-probability / convenience (not specified)	Partial	Self-report (e.g., STS+ Sexual satisfaction scale)	Limited	NR	Moderate	self-report measures; potential information and common-method bias; non-probability sampling; limited generalizability; context-specific to Iran
**Esaki K., et al. (2020) [27]**	Observational (cohort/longitudinal)	Cohort	Non-probability / convenience (not specified)	Partial (longitudinal models)	Self-report (e.g., SLE'S+LTE+ NEO-FF1)	Yes (longitudinal models)	NR	Moderate	risk of attrition and residual confounding despite adjustment; self-report measures; potential information and common-method bias; non-probability sampling; limited

									generalizability; context-specific to Japan
**Batalla V. R. D., et al. (2019) [28]**	Cross-sectional	Analytical cross-sectional	Non-probability / convenience (not specified)	Partial	Self-report (e.g., SAS+NSS)	Limited	NR	Moderate	cross-sectional design limits causal inference; self-report measures; potential information and common-method bias; non-probability sampling; limited generalizability; context-specific to the Philippines
**Turan N., et al. (2023) [29]**	Pre–post / non-randomized intervention	Quasi-experimental	Non-probability / convenience (not specified)	No / minimal	Self-report (e.g., RSA)	No / minimal	NR	High	non-randomized pre–post without control; expectancy/maturation effects; self-report measures; potential information and common-method bias; non-probability sampling; limited generalizability; context-specific to Türkiye; small,

									single-arm intervention; limited external validity
**Fadzil N.A., et al (2021) [30]**	Pre–post / non-randomized intervention	Quasi-experimental	Non-probability / convenience (not specified)	No / minimal	Self-report (e.g., PSS)	No / minimal	NR	High	non-randomized pre–post without control; expectancy/maturation effects; self-report measures; potential information and common-method bias; non-probability sampling; limited generalizability; context-specific to Malaysia; small, single-arm intervention; limited external validity
**Delgado C., et al. (2021) [31]**	Cross-sectional	Analytical cross-sectional	Non-probability / convenience (not specified)	Partial	Self-report (e.g., RAW+PWB)	Limited	NR	Moderate	cross-sectional design limits causal inference; self-report measures; potential information and common-method bias; non-probability sampling; limited

									generalizability; context-specific to Australia
**Barnett M.D., et al. (2019) [32]**	Cross-sectional	Analytical cross-sectional	Non-probability / convenience (not specified)	Partial	Self-report (e.g., SSS+ work-family balance scale)	Limited	NR	Moderate	cross-sectional design limits causal inference; self-report measures; potential information and common-method bias; non-probability sampling; limited generalizability; context-specific to USA
**Zhang L., et al. (2020) [33]**	Clinical trial	Trial / quasi-experimental	Non-probability / convenience (not specified)	Partial	Self-report (e.g., ISI+PSQI)	Yes (as reported)	NR	Moderate	trial procedures not fully detailed; randomization/blinding unclear; self-report measures; potential information and common-method bias; non-probability sampling; limited generalizability; context-specific to China; biomarker endpoints included, but

									allocation concealment not reported
**Gül, Ş. Et al. (2021) [34]**	Descriptive cross-sectional	Analytical cross-sectional	Non-probability / convenience (not specified)	Partial	Self-report (e.g., -)	Limited	NR	Moderate	cross-sectional design limits causal inference; self-report measures; potential information and common-method bias; non-probability sampling; limited generalizability; context-specific to Türkiye
**Fu C., et al. (2023) [35]**	Cross-sectional	Analytical cross-sectional	Non-probability / convenience (not specified)	Partial	Self-report (e.g., FFWC scale)	Limited	NR	Moderate	cross-sectional design limits causal inference; self-report measures; potential information and common-method bias; non-probability sampling; limited generalizability; context-specific to China
